# Metabolic dysfunction-associated steatohepatitis (MASH): intersections with type 2 diabetes and insulin resistance

**DOI:** 10.3389/fendo.2026.1901478

**Published:** 2026-08-12

**Authors:** Nick Giannoukakis

**Affiliations:** Department of Pathology, University of Pittsburgh School of Medicine, Pittsburgh, PA, United States

**Keywords:** dyslipidemia, hepatic insulin resistance, liver fibrosis, MASH, MASLD, mesenchymal stromal cells, neutrophils, sphingosine-1-phosphate

## Abstract

Metabolic dysfunction-associated fibrotic/steatotic liver disease (MASLD) and its progressive inflammatory form, metabolic dysfunction-associated steatohepatitis (MASH), are increasingly recognized as disorders of failed metabolic, immune, vascular, and reparative integration rather than as simple hepatic fat accumulation. Hepatic insulin resistance is a central pathogenic lesion because it permits persistent gluconeogenesis while maintaining or amplifying *de novo* lipogenesis, thereby linking adipose tissue dysfunction, lipotoxic hepatocyte stress, innate immune activation, endothelial injury, and fibrogenesis. This review provides an integrated framework for MASLD/MASH pathogenesis by discussing concrete preclinical and clinical examples across hepatic insulin resistance, inflammatory amplification, neutrophil biology, CXCR1/2 pathways, triglycerides, and peroxisome proliferator-activated receptor biology. Furthermore, it introduces how sphingosine-1-phosphate signaling, mesenchymal stromal cell biology, and endogenous stromal repair failure could be a functional part of this overall integration of dyslipidemia/insulin resistance-driven/conditioned MASLD/MASH. For each axis, the review summarizes observed preclinical and clinical outcomes, identifies limitations that have constrained translation, and proposes possible next steps for biomarker-enriched therapeutic development. The central conclusion is that future MASLD/MASH trials should move beyond histologic staging alone and toward biologically endotyped development programs that match metabolic, neutrophil-dominant, stromal-fibrotic, vascular, or sphingolipid-centric mechanisms to rational monotherapy or combination strategies.

## 1 Introduction

MASLD and MASH arise from the convergence of systemic metabolic stress and liver-intrinsic injury responses. Hepatic insulin resistance is a key initiating and sustaining abnormality: insulin fails to suppress hepatic glucose production while lipogenic signaling remains active, promoting triglyceride accumulation, hepatocyte stress, and inflammatory signaling (1–4). Over time, these metabolic defects become coupled to macrophage and neutrophil recruitment, sinusoidal endothelial dysfunction, hepatic stellate cell activation, and maladaptive stromal repair, establishing a feed-forward cycle that promotes fibrosis and clinical progression (5–8). This review is organized as a translational, mechanism-by-mechanism analysis. Each section provides specific preclinical and clinical examples, summarizes outcomes that have been observed, discusses limitations of the approach, and proposes next steps. The goal is to outline how MASLD/MASH therapeutic development can evolve from broad histologic enrollment toward biologically endotyped treatment selection.

## 2 Integrated mechanistic pathway framework

**Figure 1:**
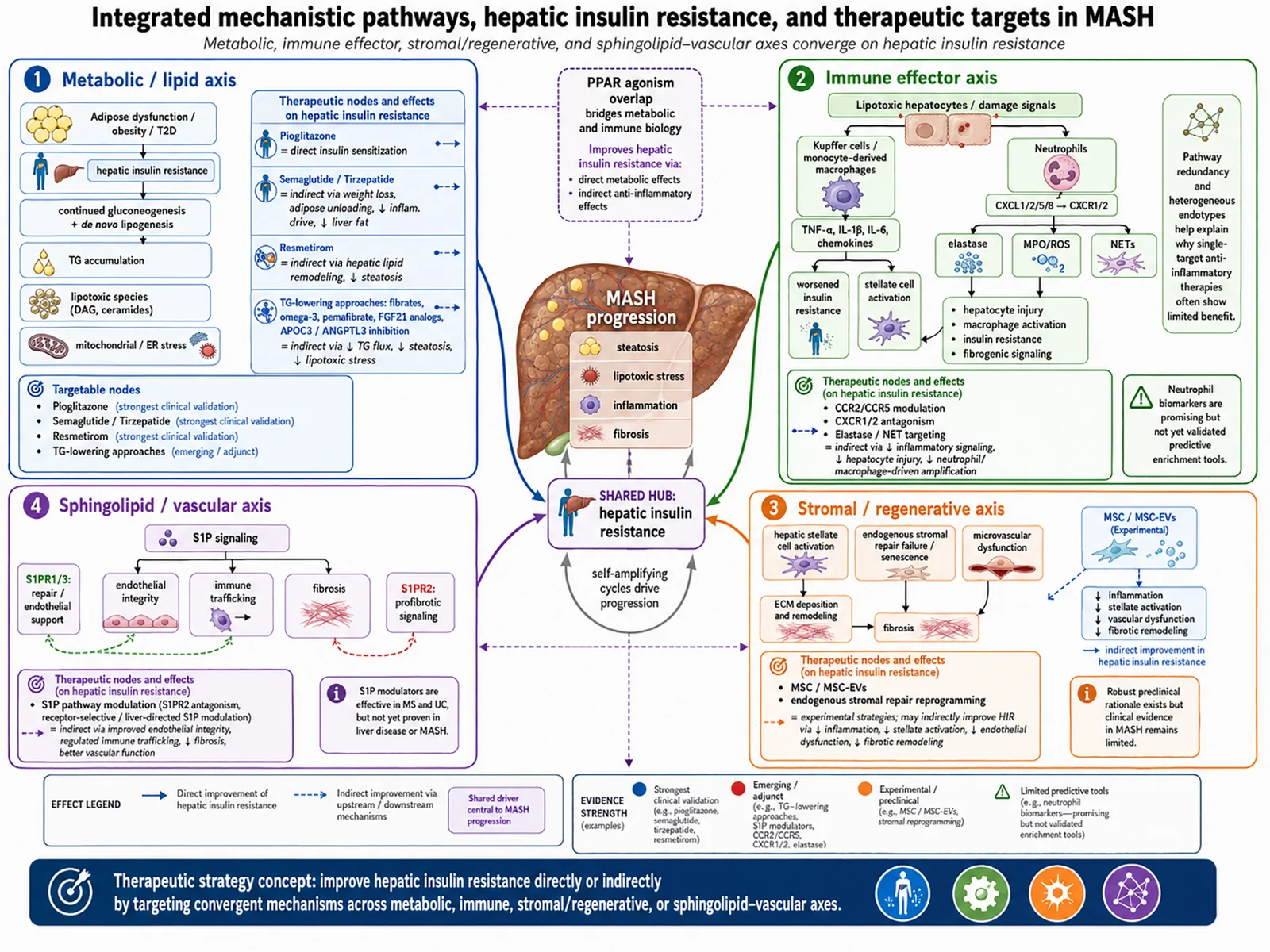
Integrated mechanistic pathways, hepatic insulin resistance, and possible therapeutic targets in MASH. The illustration summarizes how metabolic/lipid, immune effector, stromal/regenerative, and sphingolipid-vascular pathways converge on hepatic insulin resistance as a central driver of MASH progression. The metabolic/lipid axis links adipose dysfunction, obesity, type 2 diabetes, hepatic insulin resistance, persistent gluconeogenesis, de novo lipogenesis, triglyceride accumulation, lipotoxic lipid intermediates, and mitochondrial/endoplasmic reticulum stress. The immune effector axis illustrates how lipotoxic hepatocyte injury activates Kupffer cells, monocyte-derived macrophages, and neutrophils, with cytokine, chemokine, elastase, MPO/ROS, and NET-mediated amplification of hepatocyte injury, stellate-cell activation, fibrogenic signaling, and insulin signaling defects. The stromal/regenerative axis depicts hepatic stellate-cell activation, extracellular matrix deposition, microvascular dysfunction, endogenous stromal repair failure, and experimental MSC/MSC-EV approaches. The sphingolipid-vascular axis illustrates receptor-specific S1P signaling, with S1PR1/3-biased signaling framed as potentially reparative or endothelial-supportive and S1PR2 signaling as potentially profibrotic. Solid arrows indicate direct therapeutic improvement of hepatic insulin resistance, whereas dashed arrows indicate indirect effects mediated through lipid handling, inflammation, vascular integrity, or fibrotic remodeling.

MASLD/MASH can be conceptualized as a self-amplifying loop rather than a linear sequence. The metabolic/lipid axis begins with adipose dysfunction, obesity, type 2 diabetes, hepatic insulin resistance, continued gluconeogenesis, persistent *de novo* lipogenesis, triglyceride accumulation, and lipotoxic species generation. The immune effector axis is activated when lipotoxic hepatocytes release damage-associated molecular patterns, oxidized lipids, mitochondrial DNA, and extracellular vesicles that recruit or activate macrophages and neutrophils. The stromal/regenerative axis reflects the transition from reversible injury to stellate-cell activation, extracellular matrix deposition, microvascular dysfunction, senescence, and fibrosis. The sphingolipid-vascular axis links lipid metabolism to vascular integrity, immune-cell trafficking, and fibrogenesis through receptor-specific S1P biology (1–14). Triglyceride-rich lipoprotein metabolism represents an additional metabolic layer within this framework because hypertriglyceridemia reflects both increased hepatic VLDL-triglyceride production and impaired peripheral triglyceride-rich lipoprotein clearance in insulin-resistant states (Reviewed in (15)). This integrated framework also explains why single therapeutic interventions may produce incomplete responses. A metabolic therapy may reduce liver fat but leave established fibrosis or neutrophil/NET activation unresolved. Conversely, an anti-inflammatory or neutrophil-directed therapy may not succeed if insulin resistance, adipose lipid flux, and lipotoxic hepatocyte stress continue to supply upstream injury signals. Similarly, stromal or S1P-directed therapies remain biologically plausible but require careful disease-stage selection and biomarkers that identify when those pathways are dominant rather than secondary. **Figure 1** summarizes this framework and the major therapeutic target classes.

## 3 Metabolic/lipid axis: Pathobiology of hepatic insulin resistance

Hepatic insulin resistance is characterized by selective pathway impairment. Insulin signaling becomes inadequate to suppress gluconeogenesis, yet SREBP-1c-mediated lipogenesis can remain active, creating the paradox of hyperglycemia with continued hepatic lipid synthesis (1, 3). Bioactive lipid intermediates, including diacylglycerols and ceramides, impair insulin receptor signaling through PKC activation, Akt pathway disruption, mitochondrial stress, and endoplasmic reticulum stress (1, 3, 5, 7).

### 3.1 Specific preclinical and clinical examples

In high-fat diet and genetic obesity models, hepatic diacylglycerol accumulation activates PKCϵ and impairs insulin receptor kinase signaling; interventions that reduce hepatic lipid flux or restore mitochondrial oxidative capacity improve hepatic insulin sensitivity even when body weight reduction is incomplete (1, 3, 5, 7). These models demonstrate that liver fat quantity is important but insufficient: lipid species, subcellular localization, mitochondrial handling, and inflammatory context determine whether steatosis remains relatively benign or progresses to MASLD/MASH.

Clinically, insulin-sensitizing and metabolic therapies provide proof that metabolic unloading can improve liver disease activity. Pioglitazone improved steatosis, lobular inflammation, and ballooning in biopsy-proven MASH, with particularly relevant evidence in patients with prediabetes or type 2 diabetes (16–19). GLP-1 receptor agonism with semaglutide produced a significantly higher rate of MASLD/MASH resolution than placebo in a phase 2 trial, although fibrosis improvement was less consistent (20). Tirzepatide, a dual GIP/GLP-1 receptor agonist, produced MASLD/MASH resolution and fibrosis improvement signals in patients with MASLD/MASH and moderate-to-severe fibrosis, supporting the concept that profound metabolic unloading can translate into histologic benefit (21). Resmetirom, a thyroid hormone receptor-beta (THRβ) agonist, provides a complementary example of liver-directed lipid metabolism modulation, improving both MASH resolution and fibrosis in a phase 3 trial (22).

### 3.2 Limitations and next steps

Metabolic therapies often improve steatosis and inflammatory activity more consistently and earlier than fibrosis. Histologic fibrosis evolves slowly, biopsy sampling is variable, and short trials may underestimate true antifibrotic benefit or overinterpret early activity changes. In addition, metabolic therapies do not necessarily normalize neutrophil activation, macrophage polarization, endothelial dysfunction, or established extracellular matrix remodeling in advanced disease. Future trials could combine metabolic response markers, MRI-proton density fat fraction, magnetic resonance elastography or vibration-controlled transient elastography, circulating fibrosis markers, and immune/endothelial biomarkers to determine whether metabolic correction is sufficient or whether combination therapy is required. A possible informative strategy would test whether early reduction in liver fat or insulin resistance is sufficient to extinguish inflammatory and stromal signatures, or whether combination therapy is required for patients with established fibrotic or immune-dominant disease.

## 4 Triglyceride-lowering therapies at the hepatic insulin resistance interface

Hypertriglyceridemia is not merely a circulating lipid abnormality in MASLD; it reflects the same insulin-resistant metabolic network that drives hepatic fat accumulation. In insulin-resistant adipose tissue, impaired insulin suppression of hormone-sensitive lipase increases free fatty acid flux to the liver. In parallel, selective hepatic insulin resistance permits persistent gluconeogenesis while *de novo* lipogenesis remains active, increasing hepatic triglyceride synthesis, VLDL assembly, and remnant-rich dyslipidemia. In a review by Tamehri Zadeh et al. (15), an emphasis was placed on elevated plasma and intrahepatic triglyceride levels being closely associated with MASLD, often driven by insulin resistance, atherogenic dyslipidemia, and metabolic syndrome (15).

Mechanistically, triglyceride-rich lipoprotein metabolism links hepatic steatosis to systemic cardiometabolic risk. Hypertriglyceridemia arises from increased hepatic production of VLDL particles and reduced hydrolysis of VLDL and chylomicron triglycerides when lipoprotein lipase activity is impaired. ApoC-III and ANGPTL3/ANGPTL4 inhibit triglyceride-rich lipoprotein clearance, whereas statins, fibrates, ω-3 fatty acids, and FGF21 pathway activation can enhance triglyceride hydrolysis or reduce VLDL-triglyceride burden (Reviewed in (15)). Thus, triglyceride-lowering therapy can be viewed as an indirect strategy to improve hepatic insulin resistance by decreasing lipid flux, steatosis, lipotoxic intermediates, inflammatory activation, and vascular-metabolic risk.

Several therapeutic classes are relevant. Statins lower triglycerides modestly, primarily reduce ASCVD risk, and may improve endothelial, inflammatory, oxidative-stress, and fibrotic biology in MASLD, although they are not primarily MASLD/MASH-directed drugs. Fibrates activate PPARα, increase lipoprotein lipase expression, promote fatty acid transport and beta-oxidation, and may enhance hepatic insulin sensitivity through PPARα-related fatty acid oxidation and FGF21 induction. ω-3 fatty acids reduce triglyceride levels and may lower VLDL-triglyceride secretion; however, cardiovascular and hepatic outcomes vary by formulation, dose, and population, with icosapent ethyl having the strongest cardiovascular evidence among omega-3-derived approaches (Reviewed in (15)).

Emerging triglyceride-directed agents extend this concept. ApoC-III inhibitors such as volanesorsen, olezarsen, and plozasiran produce large triglyceride reductions in severe or mixed hypertriglyceridemia populations, and some studies suggest favorable changes in remnant cholesterol, non-HDL cholesterol, apoB, HDL cholesterol, or hepatic fat fraction depending on agent and population. ANGPTL3-directed strategies, including evinacumab and RNA-based approaches such as solbinsiran, improve triglyceride-rich lipoprotein metabolism and may reduce hepatic fat fraction in selected metabolic populations. FGF21 analogs such as pegozafermin and DGAT2-targeting approaches such as ervogastat or ION224 are particularly relevant to hepatic steatosis because they can reduce liver fat and metabolic injury markers, although safety, durability, histologic efficacy, and patient selection remain unresolved (Reviewed in (15)).

A key translational caveat is that triglyceride accumulation can represent both a marker of lipid excess and a buffering mechanism that packages potentially toxic fatty acids into relatively inert lipid droplets. Therefore, indiscriminate suppression of triglyceride synthesis could theoretically increase lipotoxic free fatty acids, diacylglycerols, or ceramides if lipid handling is not redirected safely. For MASH development, triglyceride-lowering therapies should therefore be framed as adjunctive or complementary metabolic strategies rather than as established stand-alone antifibrotic therapies. The most rational trials would enrich for patients with hypertriglyceridemia, remnant-rich dyslipidemia, high hepatic fat fraction, or insulin-resistant metabolic syndrome and would pair lipid endpoints with MRI-PDFF, liver enzymes, glycemic/insulin-resistance measures, inflammatory biomarkers, and fibrosis markers (Reviewed in (15)).

## 5 Immune effector axis: inflammatory redundancy and neutrophil/NET amplification

Inflammation transforms hepatic insulin resistance from a potentially reversible metabolic defect into progressive liver injury. Lipotoxic hepatocytes release damage-associated molecular patterns, oxidized lipids, mitochondrial DNA, and extracellular vesicles that activate Kupffer cells and recruit monocyte-derived macrophages (2, 4, 9, 12). These cells produce TNFα, IL-1β, IL-6, chemokines, and profibrotic mediators that interfere with insulin signaling and activate stellate cells (2, 4, 9, 12, 23, 24).

### 5.1 Why single-target anti-inflammatory therapy may fail

The inflammatory network in MASH is redundant. Cytokines, chemokines, complement, platelets, sinusoidal endothelial cells, adipose inflammation, microbial products, and metabolic stress can each sustain inflammatory recruitment and stellate-cell activation. As a result, blocking a single chemokine or cytokine pathway may reduce one component of injury without eliminating the upstream metabolic trigger or compensatory immune circuits. CCR2/CCR5 blockade with cenicriviroc reduced monocyte recruitment and fibrosis in preclinical models and advanced to clinical testing, where antifibrotic signals were observed in a subset of patients but were not strong enough to establish a definitive therapeutic role (9, 12, 25, 26). This redundancy also helps explain why histologic-stage-based enrollment can dilute pathway-specific response signals. Some patients may be macrophage-dominant, whereas others may show neutrophil/NET activation, endothelial dysfunction, adipose inflammation, complement activation, or mixed patterns. A therapy directed at one inflammatory pathway is most likely to succeed when the enrolled population is enriched for that pathway and when target engagement is demonstrated early.

### 5.2 Examples, outcomes, limitations, and possible next steps

Preclinical macrophage-targeting studies show that reducing inflammatory monocyte recruitment or reprogramming macrophage phenotype can reduce hepatic cytokine production, ALT, stellate cell activation, and fibrosis-related readouts. CCR2/CCR5 blockade with cenicriviroc reduced monocyte recruitment and fibrosis in preclinical models and advanced to clinical testing, where antifibrotic signals were observed in a subset of patients but were not strong enough to establish a definitive therapeutic role (9, 12, 25, 26).

Clinically, broad anti-inflammatory strategies have been less successful than the strength of the mechanistic rationale would predict. This probably reflects redundancy among cytokines, chemokines, complement, platelets, endothelial cells, and metabolic stress pathways. In chronic metabolic disease, systemic immunosuppression also raises tolerability and infection-risk concerns, especially in patients with diabetes, obesity, and cardiovascular comorbidity.

Inflammatory MASH is heterogeneous. Some patients are macrophage-dominant, others show neutrophil/NET activation, endothelial dysfunction, adipose inflammation, complement activation, or mixed patterns. Enrollment based solely on histologic stage can dilute response signals because a pathway-targeted therapy may be tested in patients whose disease is not driven by that pathway.

A possible practical next step is to define inflammatory endotypes using serum proteomics, circulating monocyte and neutrophil phenotyping, NET assays, spatial transcriptomics, and non-invasive fibrosis markers. Proof-of-mechanism trials should be short, biomarker-rich, and designed to show target engagement before larger histologic endpoint studies are attempted.

## 6 Neutrophils and NETs as a bridge between inflammation, insulin resistance, and fibrosis

Neutrophils are not presented here as a separate disease compartment but as immune effector cells that connect metabolic injury to insulin resistance and fibrosis. Insulin-resistant and lipotoxic livers induce CXCL1, CXCL2, CXCL5, and CXCL8/IL-8-like chemokine programs that promote neutrophil recruitment through CXCR1/2 signaling (27–30). Activated neutrophils release myeloperoxidase, neutrophil elastase, reactive oxygen species, and neutrophil extracellular traps (NETs), which can amplify hepatocyte injury, macrophage activation, stellate-cell activation, and insulin signaling defects (27–33).

### 6.1 Preclinical examples and outcomes

A key preclinical example is neutrophil elastase in diet-induced obesity. In high-fat diet-fed mice, neutrophil elastase contributes to insulin resistance by degrading insulin receptor substrate-1 and impairing insulin signaling in liver and adipose tissue; genetic or pharmacologic inhibition improved insulin sensitivity and reduced inflammatory features (28, 30). A related clinical-translational observation is the imbalance between neutrophil elastase and alpha1-antitrypsin in obesity, which links neutrophil protease burden to systemic insulin resistance and inflammatory tone (27, 29). Experimental MASH also increases hepatic NET formation, and interventions that reduce NET formation or degrade extracellular DNA attenuate inflammatory injury and fibrogenic signaling (31, 33).

Experimental steatohepatitis is accompanied by neutrophil recruitment and activation, and neutrophil effector mechanisms—including elastase release and NET formation—contribute to hepatic inflammation, insulin resistance, and liver injury. The CXCL1/CXCR2 chemokine axis provides a biologically plausible recruitment pathway for these cells, although direct evidence for CXCL1–CXCR2 blockade as a therapeutic strategy in MASH remains less extensive than the broader literature supporting neutrophil depletion or inhibition of neutrophil effector pathways (32).

### 6.2 Example clinical outcomes, limitations, and possible next steps

Clinical evidence for neutrophil-directed therapy in MASH remains largely indirect. Neutrophil-to-lymphocyte ratio, myeloperoxidase, elastase complexes, calprotectin, cell-free DNA, citrullinated histone H3, and MPO-DNA complexes have been associated with metabolic inflammation and liver disease severity in observational studies, but these biomarkers are not yet standardized as predictive enrichment tools. No neutrophil-directed drug is currently established as a MASH therapy.

Neutrophils are essential for antimicrobial host defense and tissue repair; chronic suppression can create infection risk. Murine diet models may not reproduce the age, diabetes burden, polypharmacy, microbiome variation, or chronicity of human MASH. NET biomarkers are especially sensitive to sample handling, anticoagulant choice, processing time, assay platform, and analytic definition.

One possible next step is a biomarker-enriched phase 1b/2a proof-of-mechanism trial in patients with MASH and elevated neutrophil signatures. Candidate endpoints should include safety and infection surveillance, target engagement by CXCR1/2 or neutrophil activation assays, circulating NET markers, MRI-proton density fat fraction, ALT/AST, non-invasive fibrosis markers, and exploratory spatial or transcriptomic analysis where paired biopsies are ethically justified.

## 7 PPAR and CXCR1/2 as overlapping metabolic-immune target classes

PPAR and CXCR1/2 pathways are grouped within the metabolic-immune interface rather than as an isolated section because they overlap with both insulin resistance and neutrophil biology. PPAR agonists act broadly on adipose insulin sensitivity, hepatic lipid oxidation, macrophage polarization, endothelial biology, and neutrophil activation, whereas CXCR1/2 antagonists more directly target chemokine-driven neutrophil recruitment (32, 34–36). Pioglitazone demonstrates that insulin sensitization and adipose tissue remodeling can improve MASH activity, whereas lanifibranor suggests that simultaneous PPARα, PPARδ, and PPARγ engagement may address multiple pathogenic axes (16–19, 35).

For neutrophil-directed strategies, the clinical evidence in MASH remains largely indirect. Neutrophil-to-lymphocyte ratio, myeloperoxidase, elastase complexes, calprotectin, cell-free DNA, citrullinated histone H3, and MPO-DNA complexes have been associated with metabolic inflammation and liver disease severity in observational studies, but these biomarkers are not yet standardized or validated as predictive enrichment tools. Therefore, proof-of-mechanism trials should be described cautiously as early target-engagement studies rather than definitive endotype-driven efficacy trials.

Pioglitazone, a PPARγ agonist, demonstrates that insulin sensitization and adipose tissue remodeling can improve MASH activity, but its use is limited by weight gain, edema, and heart failure concerns in susceptible patients (16–19). Lanifibranor, a pan-PPAR agonist, produced improvement in steatohepatitis activity and fibrosis-related endpoints in phase 2 testing, suggesting that simultaneous PPARα, PPARδ, and PPARγ engagement may address multiple pathogenic axes (35).

Preclinically, PPAR activation can reduce neutrophil adhesion, chemotaxis, oxidative burst, and NET formation while improving hepatic lipid handling. CXCR2 antagonism appears to attenuate neutrophil fibrosis-related injury in experimental models (32). These approaches may be especially rational for patients with inflammatory or neutrophil-dominant MASH rather than purely metabolic steatosis.

### 7.1 Example clinical outcomes, limitations, and possible next steps

For PPAR biology, clinical outcomes are encouraging but mixed. Pioglitazone remains useful in selected patients but is not ideal for broad chronic use. Lanifibranor has produced one of the stronger phase 2 histologic signals among investigational MASH therapies, but confirmatory phase 3 data, long-term cardiovascular safety, and patient-selection strategies remain important. For CXCR1/2 blockade, clinical MASH evidence is still immature; development should emphasize target engagement and enrichment rather than immediate large unselected trials.

Pan-PPAR agonism can complicate safety monitoring and mechanistic attribution because metabolic, vascular, immune, and fibrotic pathways may all change simultaneously. CXCR1/2 blockade may be too narrow if macrophage, complement, platelet, or endothelial pathways continue to recruit and activate neutrophils through alternative routes. Neither approach has yet established the ideal combination partner, duration, or biomarker-defined treatment window.

PPAR-based therapy should be prioritized for patients with insulin resistance, dyslipidemia, adipose dysfunction, and active steatohepatitis. CXCR1/2 antagonism should be tested in a neutrophil-high endotype defined by chemokines, neutrophil activation markers, or NET biomarkers. Rational combinations could pair metabolic unloading with neutrophil modulation, but sequencing and safety monitoring should be established before large phase 3 designs.

A possible practical next step is to define inflammatory and neutrophil-high states using serum proteomics, circulating monocyte and neutrophil phenotyping, NET assays, spatial transcriptomics, and non-invasive fibrosis markers. Early studies should include safety and infection surveillance, target engagement by CXCR1/2 or neutrophil activation assays, circulating NET markers, MRI-proton density fat fraction, ALT/AST, non-invasive fibrosis markers, and exploratory spatial or transcriptomic analysis where paired biopsies are ethically justified. Larger trials should await evidence that the biomarker-selected subgroup is reproducibly enriched for the intended pathway.

## 8 Thinking outside of the box: Mesenchymal stromal cells in the stromal/regenerative/immunometabolic regulator axis

Mesenchymal stromal cells (MSCs) are best understood as context-responsive immunoregulatory and trophic signaling cells rather than classical engrafting progenitors. Their therapeutic activity is mediated largely through paracrine cytokines, growth factors, extracellular vesicles, mitochondrial transfer, macrophage and neutrophil reprogramming, and endothelial stabilization (37–40).

### 8.1 Preclinical rationale

In rodent models of liver fibrosis and μASH-like injury, bone marrow-, adipose-, and umbilical cord-derived MSCs reduce ALT/AST, inflammatory cytokines, collagen deposition, alpha-smooth muscle actin expression, and histologic fibrosis. MSC extracellular vesicles reproduce many of these effects in some models, suggesting that secretome potency may be more important than durable cellular engraftment (41–44). Preclinical liver injury models show that endogenous reparative stromal responses are time-dependent. During acute injury, mobilized stromal cells and perivascular repair programs can improve endothelial stability and immune resolution. During chronic injury, the same broad stromal compartment can contribute to matrix deposition, ductular reaction, and fibrosis persistence, especially when TGFβ and mechanical stiffness dominate the tissue microenvironment (6, 8, 42, 44).

### 8.2 Clinical evidence and limitations

Clinical MSC experience is stronger in cirrhosis and immune-mediated diseases than in biopsy-proven MASH. Trials in liver cirrhosis have generally suggested short-term safety and possible improvements in liver function scores, albumin, bilirubin, ascites, or quality-of-life measures, but studies are often small, heterogeneous, and insufficiently powered for durable clinical outcomes (41, 43). Progressive fibrosis despite ongoing injury-repair signaling implies that endogenous repair is insufficient or maladaptive in advanced disease. Biomarkers of senescence, impaired angiogenic repair, and matrix turnover may eventually help identify patients in whom stromal repair is failing. For MASH specifically, there is not yet a mature clinical efficacy package demonstrating histologic MASH resolution or fibrosis regression with MSC therapy. This distinction between strong preclinical rationale and limited MASH-specific clinical evidence is important for a balanced translational interpretation.

MSC products are heterogeneous across donor, tissue source, culture conditions, passage number, cryopreservation, release assays, and potency criteria. The MASH microenvironment may impair MSC survival or reprogram MSCs toward ineffective or profibrotic phenotypes. Live-cell therapy is also difficult to justify for a common chronic disease unless the target population is advanced, high risk, and biomarker-selected. MSC extracellular vesicles or engineered secretomes may be more scalable than live-cell therapy if potency, biodistribution, and manufacturing reproducibility can be established.

### 8.3 Endogenous stromal repair

Endogenous MSC-like and perivascular stromal populations reside in bone marrow and tissue niches and are recruited to injured liver through chemotactic gradients, including S1P, CXCL12, platelet-derived growth factor, and damage-associated signals (42, 44). In early injury, these cells may support immune resolution and microvascular repair. In chronic injury, repeated exposure to TGFβ, oxidized lipids, hypoxia, and inflammatory cytokines can promote senescence, impaired angiogenic support, or profibrotic differentiation (6, 8, 42, 44).

The field needs spatially resolved maps of stromal states across MASLD, inflammatory MASH, bridging fibrosis, and cirrhosis. Single-cell RNA sequencing, spatial proteomics, lineage inference, and paired serum matrix-turnover biomarkers could define when endogenous stromal repair becomes maladaptive and whether it can be redirected therapeutically.

### 8.4 Limitations and possible next steps

Endogenous MSC-like cells are difficult to define phenotypically across liver, bone marrow, adipose tissue, and perivascular compartments. Protective and profibrotic stromal states may coexist in the same liver. Blood biomarkers may not accurately represent hepatic stromal behavior, and attempts to mobilize endogenous repair could worsen fibrosis if delivered at the wrong disease stage. The field needs spatially resolved maps of stromal states across MASLD, inflammatory MASH, bridging fibrosis, and cirrhosis. Single-cell RNA sequencing, spatial proteomics, lineage inference, and paired serum matrix-turnover biomarkers could define when endogenous stromal repair becomes maladaptive and whether it can be redirected therapeutically.

## 9 Sphingolipid-vascular axis: S1P signaling and receptor-selective concepts

S1P is a bioactive sphingolipid that links lipid metabolism, vascular biology, immune-cell trafficking, and fibrogenesis (10, 11, 13, 14). Altered sphingolipid metabolism is a hallmark of insulin resistance, and S1P signaling can be protective or pathogenic depending on receptor subtype and cellular compartment (5, 7, 11, 13). S1PR1/3 signaling may support endothelial integrity and anti-inflammatory repair, whereas S1PR2 activation has been linked to stellate-cell activation, vascular dysfunction, and fibrosis in experimental liver disease (10, 11, 13, 14).

MSCs express S1P receptors, including S1PR1, S1PR2, and S1PR3, allowing them to sense injury-associated S1P gradients (10, 14, 42, 44). In migration assays and tissue injury models, S1P gradients can direct MSC chemotaxis and improve tissue localization. However, there is no mature clinical evidence that S1P-preconditioned MSCs improve MASH outcomes. This approach remains preclinical and platform-oriented.

In humans, S1P receptor modulators have clear clinical activity in multiple sclerosis and ulcerative colitis, demonstrating that the pathway can be modulated therapeutically (45–47). However, no S1P-targeted therapy is approved for MASH, and existing agents are not optimized for liver fibrosis or hepatic insulin resistance. Their lymphocyte-trafficking effects may be either useful or undesirable depending on the target endotype. S1P receptor modulators can also affect lymphocyte counts, heart rate, blood pressure, macular edema risk, infection risk, and liver enzymes, reinforcing the need for liver-directed, receptor-selective, biomarker-guided strategies.

## 10 Integrated therapeutic outlook and future directions

MASH progression reflects failure of coordinated metabolic, immune, vascular, and regenerative programs. The strongest clinical evidence to date supports therapies that reduce hepatic fat, improve systemic metabolic risk, or directly engage hepatic lipid metabolism. Resmetirom validates liver-directed lipid signaling; semaglutide and tirzepatide validate systemic metabolic unloading; pioglitazone validates insulin sensitization in selected patients; and lanifibranor suggests that multi-axis nuclear receptor modulation can improve both inflammatory activity and fibrosis-related biology (16–22, 35). Triglyceride-lowering therapies extend this metabolic strategy by targeting VLDL-triglyceride production, triglyceride-rich lipoprotein clearance, remnant dyslipidemia, or hepatic fat accumulation, but they should presently be considered adjunctive or investigational for MASH disease modification (15). However, patients with established fibrosis, persistent inflammatory signatures, neutrophil/NET activation, stromal senescence, or S1P receptor imbalance may require combination or sequential approaches. **Table 1** summarizes mechanistic domains, targets, outcomes, limitations, next steps, and references, and **Table 2** summarizes selected therapeutic examples and translational implications with references.

**Table 1:** Mechanistic domains, targets, outcomes, limitations, next steps, and references.

Mechanistic domain	Representative examples	Observed outcomes	Key limitations	Next steps	References
Metabolic/lipid axis: hepatic insulin resistance	Pioglitazone; semaglutide; tirzepatide; resmetirom; PPAR agonism	Selective hepatic insulin resistance maintains gluconeogenesis while lipogenic signaling persists; metabolic therapies improve steatosis, MASH activity, metabolic markers, and/or fibrosis endpoints depending on agent and trial.	Fibrosis response is slower than metabolic response; biopsy variability; metabolic improvement may not fully normalize immune, endothelial, or stromal injury.	Pair metabolic endpoints with MRI-PDFF, elastography/MRE, fibrosis markers, and immune/endothelial biomarkers; consider metabolic backbone plus targeted add-on strategies.	(1, 3, 5, 7, 16–22, 35, 36)
Immune effector axis: macrophage/chemokine redundancy	Kupffer-cell and monocyte-derived macrophage pathways; CCR2/CCR5 modulation	Inflammatory recruitment and cytokine production amplify hepatic insulin resistance, hepatocyte injury, and stellate activation; clinical antifibrotic signals have been mixed.	Redundant cytokine, chemokine, complement, platelet, endothelial, and metabolic-stress circuits can bypass a single blocked pathway; systemic immunosuppression and histologic-only enrollment can dilute benefit.	Define inflammatory endotypes using proteomics, monocyte/neutrophil phenotyping, spatial biology, and non-invasive fibrosis markers before large endpoint trials.	(2, 4, 9, 12, 25, 26)
Neutrophil/NET interface	Neutrophil elastase inhibition; NET modulation; CXCR1/2 or CXCR2 antagonism	Neutrophil elastase and NETs can worsen insulin signaling, hepatocyte injury, macrophage activation, and fibrogenic signaling in experimental systems.	Host-defense risk; murine models incompletely reproduce human MASH; NET and neutrophil biomarkers are promising but not validated as predictive enrichment tools.	Use cautious, biomarker-rich early studies focused on safety, target engagement, and pathway modulation before larger efficacy trials.	(27–33)
PPAR/CXCR1/2 overlap	Pioglitazone; lanifibranor; CXCR1/2 antagonism	PPAR agonism bridges metabolic and immune biology; CXCR1/2 blockade more directly targets neutrophil recruitment.	Mechanistic attribution can be difficult for pan-PPAR therapy; CXCR1/2 blockade may be too narrow if redundant inflammatory pathways remain active.	Group therapeutic development by metabolic-dominant versus immune-effector/neutrophil-high biology and test rational sequencing or combinations.	(16–19, 32, 34–36)
Stromal/regenerative axis: MSC therapy	Bone marrow-, adipose-, and umbilical cord-derived MSCs; MSC extracellular vesicles	Preclinical liver injury and fibrosis models show reductions in inflammation, ALT/AST, stellate activation, collagen deposition, and fibrosis-related readouts.	MASH-specific clinical efficacy remains limited; products are heterogeneous across donor, source, culture, passage, cryopreservation, and potency assays.	Separate robust preclinical rationale from limited clinical evidence; develop potency assays tied to neutrophils, macrophages, stellate cells, endothelium, and hepatocyte stress.	(37, 39–43)
Endogenous stromal repair	Perivascular and bone marrow stromal responses; MSC-like stromal states	Early stromal responses may support immune resolution and microvascular repair, whereas chronic injury can drive senescence, matrix deposition, and fibrosis persistence.	Human stromal states are poorly defined; protective and profibrotic states may coexist; blood biomarkers may not reflect hepatic stromal behavior.	Map stromal states with single-cell RNA sequencing, spatial proteomics, lineage inference, and paired serum matrix-turnover biomarkers.	(6, 8, 37–44)
S1P/MSC interactions	S1P gradients; S1PR1/3-biased or S1PR2-suppressed MSC concepts	S1P can influence MSC chemotaxis, survival, angiogenic signaling, and immunoregulatory outputs in experimental systems.	Clinical MASH data are absent; MSC receptor expression varies with donor features, culture conditions, inflammatory priming, and passage number.	Compare unmodified MSCs, receptor-biased MSCs, and MSC-EVs in standardized MASH models with hepatic homing, fibrosis, and insulin-signaling endpoints.	(10, 14, 42, 44)
Sphingolipid-vascular axis	S1PR modulators; S1PR2 antagonism concepts	S1P signaling links lipid metabolism, vascular biology, immune-cell trafficking, and fibrogenesis; S1PR modulators validate druggability in MS/UC.	No S1P-targeted therapy is approved for MASH; systemic immune, cardiovascular, ocular, infection, and liver-enzyme effects require caution.	Frame S1P approaches as investigational for MASH; prioritize receptor-selective, liver-directed, biomarker-guided strategies.	(10, 11, 13, 14, 45–47)
Metabolic/lipid axis: triglyceride-rich dyslipidemia and TG-lowering therapy	Statins; fibrates; omega-3 fatty acids/icosapent ethyl; pemafibrate; ApoC-III inhibitors; ANGPTL3 inhibitors; FGF21 analogs; DGAT2-targeting approaches	TG lowering may reduce VLDL-TG burden, remnant-rich dyslipidemia, hepatic fat fraction, liver enzymes, and selected metabolic injury markers; some emerging agents produce large TG reductions or hepatic fat reductions in selected populations.	MASH disease modification is not established; TG storage can buffer fatty acid lipotoxicity; hepatic and cardiovascular outcomes vary by agent, formulation, population, duration, and endpoint.	Use as adjunctive/complementary metabolic strategy; enrich trials for hypertriglyceridemia, remnant-rich dyslipidemia, high MRI-PDFF, and insulin-resistant metabolic syndrome; include lipid, imaging, inflammatory, glycemic, and fibrosis endpoints.	(15)

**Table 2:** Selected therapeutic examples and translational implications for MASLD/MASH.

Therapeutic class or pathway	Example(s)	Clinical/preclinical outcome summary	Development implication	References
Insulin sensitization	Pioglitazone	Improved steatohepatitis activity in biopsy-proven MASH, especially relevant in prediabetes or type 2 diabetes.	Direct insulin-sensitizing proof-of-concept, but weight gain, edema, and heart-failure risk limit broad chronic use.	(16–19)
Incretin-based metabolic unloading	Semaglutide; tirzepatide	Improved MASH resolution and weight/metabolic endpoints; fibrosis responses vary by agent, dose, duration, and trial population.	Strong metabolic backbone for combination strategies; hepatic insulin resistance may improve indirectly through weight loss, reduced adipose flux, and reduced liver fat.	(20, 21)
Liver-directed lipid metabolism	Resmetirom	Phase 3 improvement in MASH resolution and fibrosis endpoints.	Validates hepatic lipid signaling as a disease-modifying axis and may indirectly improve insulin resistance through hepatic lipid remodeling and reduced steatosis.	(22)
Pan-PPAR modulation	Lanifibranor; pioglitazone	Clinical histologic benefit with selected PPAR agents, including phase 2 signals for lanifibranor.	Bridges metabolic, inflammatory, vascular, and fibrotic biology; requires safety monitoring and clearer patient-selection strategies.	(16–19, 34–36)
Macrophage/chemokine modulation	CCR2/CCR5 blockade	Preclinical rationale and mixed clinical antifibrotic signals.	Single-target anti-inflammatory therapy may fail because of pathway redundancy and unselected enrollment.	(9, 12, 23–26)
Neutrophil recruitment/activation	CXCR1/2 antagonism; elastase/NET targeting	Preclinical reduction in insulin resistance, neutrophil recruitment, injury, and fibrosis-related readouts.	Requires cautious neutrophil-high enrichment, infection surveillance, target engagement, and recognition that predictive biomarkers are not yet validated.	(27–33)
MSC/MSC extracellular vesicles	Live MSCs; extracellular vesicles	Preclinical reductions in inflammation and fibrosis; MASH-specific clinical efficacy remains limited.	Promising but experimental for MASH; requires potency assays, manufacturing control, biodistribution data, and endotype selection.	(37–41, 43)
S1P receptor biology	S1PR modulators; S1PR2 antagonism concepts	Approved S1PR modulators validate the pathway in MS/UC; liver and MASH applications remain investigational.	Receptor selectivity, liver targeting, immune/cardiovascular safety, and biomarker-defined pathway dominance are essential.	(10, 11, 13, 14, 42, 44–47)
Triglyceride-lowering therapies	Statins; fibrates; ω-3 fatty acids/icosapent ethyl; pemafibrate; volanesorsen/olezarsen/plozasiran; evinacumab/solbinsiran; pegozafermin; DGAT2-targeting approaches	Current and emerging agents can lower circulating TGs, enhance TG-rich lipoprotein clearance, reduce VLDL/remnant burden, or reduce hepatic fat fraction; ApoC-III and ANGPTL3 approaches can produce large TG reductions, while FGF21 and DGAT2-directed approaches have liver-fat relevance.	Rational adjunct to weight loss, insulin sensitization, and anti-inflammatory strategies, especially in hypertriglyceridemic or remnant-rich dyslipidemic MASLD; long-term MASH histologic and antifibrotic efficacy remains to be proven.	(15)

### 10.1 Possible next-generation development schema

A next-generation MASH program could proceed in four steps. First, classify patients by dominant biology using MRI-proton density fat fraction, elastography or magnetic resonance elastography, metabolic markers, inflammatory proteomics, neutrophil/NET assays, sphingolipidomics, and matrix-turnover markers. Second, select therapy based on the dominant pathway while recognizing that predictive endotypes remain incompletely validated. Third, use short, cautious proof-of-mechanism trials to confirm target engagement and early pathway correction rather than overclaiming efficacy. Fourth, advance only biologically responsive and clinically plausible subgroups into longer histologic or outcomes trials.

## 11 Conclusion

MASLD/MASH is not simply fat accumulation in the liver, nor is it only fibrosis. It is a failure of integrated metabolic, immune, vascular, and reparative control. Current clinical success with resmetirom and incretin-based therapies validates metabolic and hepatic lipid biology, but it does not fully address inflammatory, neutrophil, stromal, and sphingolipid axes that may drive progression in specific patient subsets. The next stage of MASLD/MASH therapeutic development should therefore use mechanism-defined groupings, fit-for-purpose biomarkers, cautious target-engagement studies, and rational combinations to match therapy to dominant disease biology.

## Data Availability

Publicly available datasets were analyzed in this study. This data can be found here: No new data were generated or analyzed for this review article.
