# Supplementary material for: Metabolic dysfunction-associated steatohepatitis (MASH): intersections with type 2 diabetes and insulin resistance
Source: Front Endocrinol (Lausanne). 2026 Aug 12;17:1901478. doi: 10.3389/fendo.2026.1901478 (PMC13506278; doi:10.3389/fendo.2026.1901478)
Supplement: Supplementary file 1 [file DataSheet1.pdf]

Summary:

This structured narrative review used targeted searches of PubMed/MEDLINE and Europe PMC, supplemented by citation chaining and targeted journal searches, to identify peer-reviewed mechanistic, preclinical, translational, and clinical literature published from January 2021 through July 25, 2026. Evidence was selected purposively for relevance to hepatic insulin resistance, inflammatory amplification, neutrophil biology, CXCR1/2 pathways, triglycerides, peroxisome proliferator-activated receptor biology, sphingosine-1-phosphate signaling, and mesenchymal stromal cell biology and was appraised using design-appropriate methodological domains. Because the review was explanatory and hypothesis-generating rather than exhaustive or designed to estimate pooled effects, findings were synthesized narratively. For each axis, the review summarizes observed preclinical and clinical outcomes, identifies limitations that have constrained translation, and proposes possible next steps for biomarker-enriched therapeutic development. The central conclusion is that future MASLD/MASH trials should move beyond histologic staging alone and toward biologically endotyped development programs that match metabolic, neutrophil-dominant, stromal-fibrotic, vascular, or sphingolipid-centric mechanisms to rational monotherapy or combination strategies.

Reporting framework and methodological limitations:

PRISMA 2020 provides reporting guidance for systematic reviews and includes a flow diagram that requires exhaustive record identification, de-duplication, screening, retrieval, and exclusion counts [28]. Applying such a diagram retrospectively to this structured narrative review would inaccurately imply a protocol-driven, exhaustive selection process. Accordingly, the manuscript does not present a PRISMA flow diagram. Instead, it reports the information sources, search concepts, eligibility criteria, selection approach, evidence appraisal, and synthesis methods used. The limitations of this approach include possible selection bias, single-author screening and appraisal, lack of protocol registration, absence of duplicate data extraction, and no formal study-level risk-of-bias or certainty-of-evidence rating. Conclusions should therefore be interpreted as an evidence-informed translational synthesis rather than as an exhaustive estimate of comparative treatment effects.

Methods: structured narrative review approach

<u>Review design and scope</u>:

This article was designed as a structured narrative review with a systematic and reproducible literature-identification framework, not as a systematic review or meta-analysis of a narrowly defined intervention, exposure, or outcome. Its purpose was to integrate heterogeneous mechanistic, preclinical,

translational, and clinical evidence across multiple interacting domains of MASLD/MASH biology and to identify translational implications, limitations, and testable development strategies.

Information sources and search strategy:

PubMed/MEDLINE and Europe PMC were searched for English-language, peer-reviewed literature published from January 1, 1995 through July 25, 2026. Database searches were supplemented by backward and forward citation chaining from highly relevant reviews, trials, and mechanistic studies and by targeted searches of journal and publisher websites. Search concepts included the disease terms "metabolic dysfunction-associated steatotic liver disease," "metabolic dysfunction-associated steatohepatitis,", "MAFLD", "MASLD," "MASH," "nonalcoholic fatty liver disease," "NAFLD," "nonalcoholic steatohepatitis," and "NASH." These were combined with terms for the principal mechanistic and therapeutic domains: "hepatic insulin resistance," "diacylglycerol," "ceramide," "inflammation," "macrophage," "Kupffer cell," "neutrophil," "neutrophil extracellular trap," "CXCR1," "CXCR2," "triglyceride," "ApoC-III," "ANGPTL3," "FGF21," "DGAT2," "PPAR," "pioglitazone," "lanifibranor," "mesenchymal stromal cell," "mesenchymal stem cell," "extracellular vesicle," "sphingosine-1-phosphate," "S1P," "S1PR," "semaglutide," "tirzepatide," "resmetirom," and "cenicriviroc." Terms were adapted to the syntax of each source.

Eligibility criteria and evidence selection:

Eligible publications included randomized and nonrandomized clinical studies, systematic reviews and meta-analyses, human observational studies, preclinical in vivo or mechanistic studies, and authoritative mechanistic reviews that directly informed at least one prespecified domain. Studies were prioritized when they provided clinically relevant efficacy or safety outcomes, direct mechanistic evidence, target-engagement or biomarker data, or a clear link between metabolic dysfunction and inflammatory, stromal, vascular, or sphingolipid biology. Conference abstracts without a full peer-reviewed report, editorials, non-English publications, duplicate reports, studies without direct relevance to the review questions, and articles lacking sufficient methodological or outcome detail were excluded.

Titles and abstracts were reviewed first, followed by full-text evaluation of publications considered potentially informative. Selection was iterative and purposive: studies were chosen to represent the strongest and most current evidence for each mechanistic domain, clinically important positive or negative findings, and major translational limitations. Reference lists were re-examined until no additional high-priority evidence was identified. Because this was a narrative evidence synthesis rather than an exhaustive systematic review, no claim of complete record capture is made and a formal record-level screening log was not used.

Data extraction, evidence appraisal, and synthesis:

For each included publication, the author extracted the study design, model or population, intervention or mechanistic exposure, principal outcomes, translational relevance, and major limitations. Evidence was appraised using design-appropriate domains. Randomized trials were considered with respect to randomization, allocation concealment, masking, attrition, endpoint definition, and consistency of prespecified analyses. Systematic reviews and meta-analyses were considered with respect to search breadth, eligibility criteria, risk-of-bias assessment, heterogeneity, and publication bias. Observational studies were evaluated for selection bias, confounding, measurement validity, and generalizability. Preclinical studies were considered with respect to model relevance, randomization and blinding where reported, sample-size justification, replication, and concordance with human biology. Narrative and mechanistic reviews were evaluated for recency, breadth, balance, and traceability to primary evidence.

Quality judgments were used to calibrate the strength of interpretation rather than to generate a pooled score or automatically exclude evidence. Greater weight was assigned to well-conducted randomized trials, systematic reviews, replicated mechanistic findings, and studies with direct human translational relevance. Findings supported only by a single model, indirect biomarkers, small samples, post hoc analyses, or heterogeneous products were explicitly described as preliminary or hypothesis-generating. Owing to substantial heterogeneity in populations, models, interventions, and endpoints, no meta-analysis or formal certainty-of-evidence grading was undertaken. Evidence was synthesized narratively within the prespecified metabolic/lipid, immune-effector, neutrophil/NET, PPAR/CXCR1/2, stromal/regenerative, and sphingolipid-vascular domains.
